# Molecular Aspects of Senescence and Organismal Ageing—DNA Damage Response, Telomeres, Inflammation and Chromatin

**DOI:** 10.3390/ijms22020590

**Published:** 2021-01-08

**Authors:** Natalia Sławińska, Renata Krupa

**Affiliations:** Laboratory of Medical Genetics, Faculty of Biology and Environmental Protection, University of Lodz, 90-236 Lodz, Poland; natalia.slawinska@edu.uni.lodz.pl

**Keywords:** ageing, senescence, DNA damage response, telomeres, inflammation, chromatin, SASP

## Abstract

Cells can become senescent in response to stress. Senescence is a process characterised by a stable proliferative arrest. Sometimes it can be beneficial—for example, it can suppress tumour development or take part in tissue repair. On the other hand, studies show that it is also involved in the ageing process. DNA damage response (DDR) is triggered by DNA damage or telomere shortening during cell division. When left unresolved, it may lead to the activation of senescence. Senescent cells secrete certain proteins in larger quantities. This phenomenon is referred to as senescence-associated secretory phenotype (SASP). SASP can induce senescence in other cells; evidence suggests that overabundance of senescent cells contributes to ageing. SASP proteins include proinflammatory cytokines and metalloproteinases, which degrade the extracellular matrix. Shortening of telomeres is another feature associated with organismal ageing. Older organisms have shorter telomeres. Restoring telomerase activity in mice not only slowed but also partially reversed the symptoms of ageing. Changes in chromatin structure during senescence include heterochromatin formation or decondensation and loss of H1 histones. During organismal ageing, cells can experience heterochromatin loss, DNA demethylation and global histone loss. Cellular and organismal ageing are both complex processes with many aspects that are often related. The purpose of this review is to bring some of these aspects forward and provide details regarding them.

## 1. Introduction

Organismal ageing is a complex process that depends on many interconnecting factors. It is characterised by a gradual accumulation of physical and molecular dysfunction, tissue degradation and diminishing organ function. It increases the risk of developing age-related diseases and ultimately ends in death [1]. Cellular ageing, also referred to as senescence, is one of the cellular responses to various stimuli, such as stress, DNA damage, telomere shortening, or substances secreted from other cells. Senescent cells are characterised by complete cessation of proliferation [2] and certain morphological changes—cells become larger and flat in shape [1]. The role of senescence in tumour suppression, tissue repair and organismal ageing is being researched. Senescence can be reversed during tumour suppression and tissue repair but not when it is linked to organismal ageing [3]. This review will focus on certain aspects of cellular and organismal ageing processes and the way they affect each other.

## 2. Cellular Ageing

### 2.1. DNA Damage Response in Ageing Cells

DDR (DNA damage response) is a molecular pathway initiated by cells in response to DNA damage. If not repaired, DNA damage can lead to various mutations and destabilise translation and transcription [4].

The most detrimental type of DNA damage is DSBs (double-strand breaks). Unrepaired DSBs can be the cause of senescence in proliferating cells [5,6] There are two main cellular systems of DSB repair—HR (homologous repair) and NHEJ (non-homologous end joining) [7]. NHEJ can be further divided into canonical NHEJ (c-NHEJ) and alternative NHEJ (alt-NHEJ). HR relies on sister chromatids to repair breaks in a relatively error-free way, but it can be implemented only when sister chromatids are present, so it is used mainly in S and G2 phases of the cell cycle [8]. NHEJ, on the other hand, does not require a homologous sequence to repair DSBs, although joined ends sometimes include short homologous sequences of less than~10 bp—this is called microhomology [9]. cNHEJ is the main way of repairing DSBs in G1 phase. Additionally, studies have shown that, contrary to the expectations, cNHEJ, and not HR, is also the most frequently used DSBs-repairing pathway during S and G2 phases [8]. cNHEJ pathway is repressed in the telomere area. While cNHEJ is unable to repair double-strand breaks that occur on telomeres, alt-NHEJ does not have such restrictions. It relies on microhomology to repair DSBs, so telomeres are perfect targets for this pathway, due to being comprised of high number of repetitive sequences [10]. Unfortunately, alt-NHEJ is an error-prone pathway and can facilitate chromosome fusions, extremely detrimental to the cell [10,11]. 

**Figure 1:**
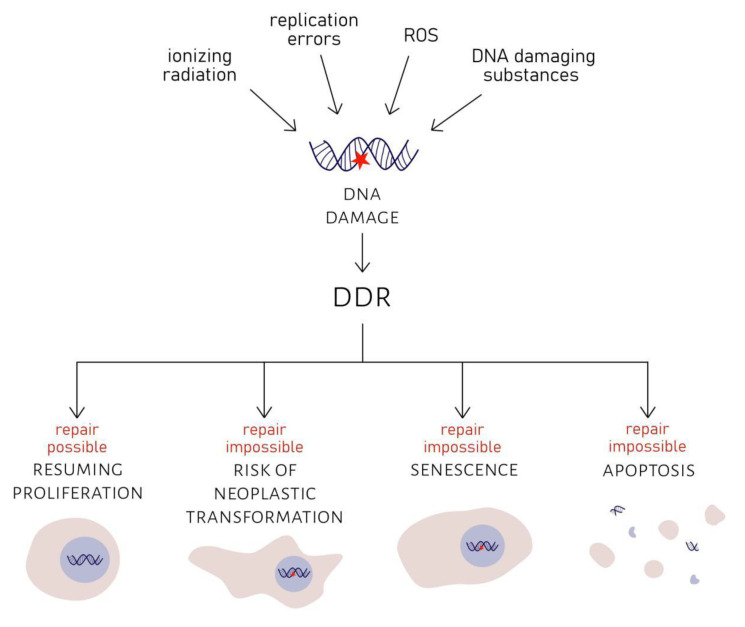
Consequences of DNA damage. DNA damage, that can be caused by ionizing radiation, replication errors, ROS (reactive oxygen species) or various DNA damaging substances, induces DDR (DNA damage response). Proliferation is halted. If damage can be successfully repaired, cell can resume proliferation. If DNA damage cannot be repaired, the cell enters senescence or apoptosis. If none of these three outcomes take place, the cell risks undergoing neoplastic transformation.

DDR pathway consists of numerous proteins. After formation of DSB, MRN complex (MRE11, RAD50, NBS1) detects DNA damage. ATM and ATR kinases are recruited to the site of damage and phosphorylate histone variant H2AX. H2AX phosphorylation is required for the assembly of checkpoint proteins and DNA repair factors—53BP1, MDC1/NFBD1, NBS1 and others. γH2AX promotes the activation of Chk1 and Chk2 transducer kinases (by phosphorylation). Activated Chk1 and Chk2 relay the signal to p53/p51 proteins [12]. If repair is achievable, DDR foci disappear usually within 24 h, and the cell can resume proliferation. However, if repair is impossible, the cell is left with persistent DDR foci [13,14]. Such foci do not show any signs of DNA repair. They are characteristic of the senescent phenotype and can be the cause of senescence themselves [13]. Alternatively, apoptosis can be another outcome for cells with permanently damaged DNA [6]. Such mechanisms, if they work properly, ensure that dysfunctional genetic information is not passed down to progeny cells; hence, genome integrity can be preserved. They can also prevent neoplastic transformations, which could occur if cells with damaged DNA continued proliferation [4,13]. The consequences of DNA damage are summarised in Figure 1.

### 2.2. Role of Telomere Damage in Senescence

Telomeres are nucleoprotein complexes located at the ends of linear chromosomes [15,16]. DNA components contain two elements—a double-stranded fragment consisting of repetitive TTAGGG sequences identical for each vertebrate, including humans and 3’ single stranded G-rich overhang [17,18,19].

Telomeres contain a distinctive structure called t-loop. A t-loop is formed by the insertion of 3’ end of a single DNA strand between two strands of the double helix [20,21]. Creation of this structure and interaction with the protein compound protect chromosome ends’ fusions and avoid their recognition as the double strand break’s DNA ends by DNA repair systems. 

Protein compound includes a telomerase and a complex of six proteins, which form shelterin [22]. Those proteins are TRF1, TRF2, TIN2, RAP1, TPP1 and POT1 in humans. TRF2 is directly responsible for t-loop formation [20]. Shelterin binds both single and double stranded telomeric DNAs. The part of shelterin that consists of four proteins TRF1, TRF2, TIN2, RAP1 binds double-stranded parts of telomeric DNA while POT1 and TPP1 heterodimer is responsible for binding single-stranded overhang of telomeric DNA [23,24,25,26,27]. An ectopic tethering of TRF2 to internal DSB in mice fibroblasts prevented DSB repair [28]. TRF2 inhibition triggers recruitment of DNA damage response proteins [22]. Inhibition of TRF2 in human lung fibroblasts caused chromosome end fusions. The result was premature senescence resembling replicative ageing [29].

Telomere length varies with species [30,31]. In humans, the length is between 5000 and 15,000 nucleotides; in mice, the telomeres are longer and have a much faster shortening rate [32]. Generally smaller, shorter-lived species have longer telomeres and expressing telomerase, which is silenced in somatic cells of species having shorter human-like telomeres [30,33]. 

Telomere elongation is another process regulated by shelterin [22]. TRF2 overexpression in human IMR90 fibroblasts accelerated telomere shortening. IMR90 cells were infected with TRF2-expressing retrovirus. Telomere loss in these cells was accelerated by 50–80% (in comparison to cells infected with the retrovirus that did not express TRF2) [34]. Longer telomeres contain more shelterin, hence more TRF2. It means that long telomeres are characterised by lower telomerase activity, which leads to telomere shortening [22]. Despite this, TRF2 overexpression does not cause premature senescence, but, on the contrary, it postpones it. This is the effect of the protective properties of TRF2, which prevent chromosome fusion. Owing to that, entering a senescence state is stalled [34].

All shelterin components are involved in telomere length regulation as they take a part in telomerase activity regulation, protection of telomeres from degradation or by preventing unnecessary activation of DNA repair processes (see reviews [22,35], for more details). 

Telomerase is a DNA polymerase that consists of two units, namely, reverse transcriptase (TERT) and RNA component (TERC). It synthesises TTAGGG repeats on the ends of telomeres, thus preventing chromosome shortening and, sometimes, even extending them [36].

The role of telomeres in cellular ageing is twofold:

(1) If double-strand breaks that appear on telomeres due to random factors cannot be repaired, DDR proteins remain permanently attached to the damage site and the cell enters the state of senescence. These sites of DNA damage are referred to as telomere associated foci (TAF). This is one of the causes of ageing of cells that are not proliferating (differentiated or quiescent). DNA damage repair within telomeres is suppressed by TRF2. This mechanism is indispensable to the cell as it prevents telomere fusion due to NHEJ. However, TRF2 together with Rap1, do not allow regular DSBs that can appear within telomeres due to DNA damaging factors to be repaired [5,28]. In mammalian cells, ectopic insertion of TRF2 in the vicinity of DSB leads to the inability of repairing them and to persistent DDR activation [28]. The presence of TAF is independent of telomere length and telomerase activity; however, it does depend on the age of the organism. The number of TAF in mice hepatocytes and enterocytes increased exponentially with age [37].
(2) DNA damage response is triggered as a result of telomere shortening in proliferating cells. A permanent loss of the ability to divide due to this reason is called replicative ageing. The process of replicative ageing was described for the first time by Hayflick and Moorhead in 1961 when they noticed the limit of divisions that fibroblasts were able to undergo. After going through approximately sixty divisions, cells stopped proliferation and entered a state that was called senescence. The limit of divisions that cells were capable of due to telomere attrition was called the Hayflick limit. Telomere shortening as a result of replicative ageing causes uncapping of a single-stranded fragment on the end of a telomere. DDR factors begin to localise to the uncapped ends, and the cell enters senescence or undergoes apoptosis [38,39]. When the telomere reaches its critical length (under 6000–8000 base pairs), shelterin that protected it is lost [40]. DDR factors such as 53BP1, γ-H2AX, RAD17, ATM and Mre11 localise at these unprotected telomeres [39]. Along with consecutive cell divisions, the number of γ-H2AX foci on telomeres rises from 10% to 72% [40]. These changes are distinctive for double-strand breaks. This kind of telomere damage cannot be repaired. Thus, DDR foci persist, and cell enters the state of senescence [38].

### 2.3. Senescence-Associated Secretory Phenotype

Increased secretion of certain proteins during senescence is referred to as senescence-associated secretory phenotype (SASP) [41].

SASP can have a negative influence on neighbouring cells. As Shelton et al. (1999), Coppé et al. (2008) and Childs et al. (2015) inform, the compounds included in SASP include, among others:

- Chemokines and cytokines which cause inflammation and regulate immune response (e.g., IL-6, IL-8 and CCL);
- Growth factors (e.g., GRO, HGF and IGFBP);
- Metalloproteinases which degrade the extracellular matrix;
- Reactive oxygen species (ROS) and nitrogen oxide (I) [41,42,43].

The variety of secreted substances depends on cell type—for example, senescent retinal pigment epithelium (RPE) does not secrete inflammation regulating factors as opposed to senescent human fibroblasts [41]. 

On the other hand, SASP can also have a positive impact. Some substances included in SASP initiate the rejuvenation of damaged tissues. Interleukins IL-6 and IL-8 contribute to the initiation of senescence caused by oncogenes and arrest divisions of neoplastic cells at an early stage. Nineteen out of twenty human colon adenomas contained IL-8-positive groups of cells that also had antigen Ki-67 absent from their nuclei, which indicates a proliferative arrest. Surrounding IL-8-negative cells continued divisions [44]. On the contrary, SASP can also play a negative role in carcinogenesis. Senescent fibroblasts cultured in close proximity to human keratinocytes, preneoplastic epithelium cells and breast cancer MDA231 cells accelerated the division of preneoplastic cells 2-4-fold, and 3-7-fold in case of neoplastic cells [45]. In breast cancer patients, the concentrations of IL-6 and IL-8 were significantly higher than in controls, and their levels increased in accordance with the clinical tumour stage [46].

SASP proteins start to appear within the first 4–7 days after a cell enters senescence caused by DNA damage; therefore, they cannot simply be considered a part of the DDR pathway [41]. A high dose of X radiation (10 Gy) caused an increase in IL-6 and IL-8 secretion 5 – 6-fold during a period of 2 to 4 days, and even higher in 3 to 5 days. It was also established that the secretion of inflammatory cytokines could be a direct effect of persistent DDR foci presence, and not senescence per se [47].

Sirtuin 1 (SIRT1) is one of the factors which regulate SASP. It is a NAD^+^ dependant deacetylase that controls many biological processes by means of deacetylation of transcription factors, histones, repair enzymes and other proteins [48]. Furthermore, SIRT1 binds to DSBs and is involved in their repair [49]. Sirtuin 1 lowers the expression of genes encoding IL-6, IL-8 and other SASP proteins. Decreasing SIRT1 levels in human fibroblasts led to an increase in IL-6 and IL-8 expression [48]. SIRT1 concentration is affected by oxidative stress. ROS deplete NAD^+^ that is necessary for the activation of PARP-1 (a DNA repair enzyme), which regulates SIRT1 [50]. Reactive oxygen species cause significant depletion of satellite-bound SIRT1. More than 90% of promoters lost SIRT1 previously associated with them in cells subjected to oxidative stress. It was caused by the relocation of SIRT1 to double-strand breaks that came into existence in the wake of the oxidative stress. As a result, sequences previously suppressed by SIRT1 could be expressed [49].

### 2.4. Changes in the Chromatin Structure of Senescent Cells

Heterochromatin of senescent cells undergoes certain structural changes. One of such changes is the formation of SAHFs, i.e., senescence-associated heterochromatin foci. SAHFs are not transcriptionally active; therefore, their formation alters the transcriptome of the cell [51]. SAHF is not formed in the regions of constitutive heterochromatin such as telomeres or centromeres [51,52,53]. In a study conducted on senescent WI-38 fibroblasts, individual chromosomes were condensing, which resulted in the formation of individual SAHF [52]. Major propagators of SAHF formation are the following chromatin regulators: HIRA (histone repressor A) and ASF1a (anti-silencing function 1a). PML nuclear bodies are structures located in the nucleus which become enlarged during senescence. Recruitment of HIRA and ASF1a to PML nuclear bodies in senescent cells led to the formation of SAHF that contained the H2A histone variant—macroH2A [54]. SAHF also contains other proteins typical of transcriptionally inactive chromatin, namely, H3 histone methylated on lysine 9 (H3K9Me) and HP1 [51,53]. The formation of SAHF is the reason for the stability of a proliferative arrest during senescence and the lack of reaction to mitogenic signalling. SAHF causes stable suppression of E2F transcription factor target genes. Their expression is necessary for the cell to transition from G_1_ to S phase. These genes are silenced in SAHF by the retinoblastoma (Rb) protein family. Rb controls the heterochromatin structure in senescent cells and is responsible for senescence stabilisation [51].

Another modification of chromatin in senescent cells is the loss of linker histone H1 in SAHFs (this occurred in senescent fibroblasts of MRC-5 and IMR-90 cell lines but not BJ cell line). Synthesis of new H1 histones does not occur as a result of post-translational repression. Ectopic expression of H1 histone in WI-38 cells increased its quantity in chromatin by an insignificant amount and did not prevent SAHF formation or entering senescence [52].

Another change that cells undergo after entering senescence is the loss of lamin B1 [55,56]. Lamins form a nuclear lamina on the inner side of the nuclear membrane. They influence not only the structure and shape of the nucleus but also chromatin structure and gene expression [57]. Lamin B1 loss occurred in the span of two days since exposure to ionising radiation of 10 Gy [55]. The quantity of lamin B1 decreased by approximately 80–90% in BJ senescent cells. This process does not occur when senescence is not the cause for arrested cell divisions. An immediate cause for lamin B1 loss is a decreased quantity and stability of LMNB1 gene transcripts—the number of transcripts declined by approximately 95% [55,58]. Lamin B1 gene silencing can induce senescence in cells [58]. These attributes suggest that lamin B1 loss could find application as a marker of senescence [55].

One more process that takes place during senescence is chromatin decondensation at centromeres and satellites, i.e., SADS (senescence-associated distension of satellites). SADS takes place at an early stage after a cell becomes senescent, preceding SAHF formation. SADS differs from other types of DNA decondensation. In spite of decondensation, increased expression of satellite RNA was not observed. Histone H1 loss is not the cause of SADS, as 64% of cells exhibiting SADS still had high levels of H1. Nevertheless, it could potentially be linked with lamin B1 loss. 95% of cells with normal lamin B1 levels had condensed DNA at satellites, whereas in cells with lowered levels of lamin B1, 75% of satellites were condensed [59].

## 3. Organismal Ageing

### 3.1. The Role of Telomeres in Organismal Ageing

Studies frequently show that there is a link between telomere length and the age of an organism [60,61]. Telomeres gradually shorten with age; the speed of attrition differs significantly depending on cell type, frequency of cell divisions and telomerase activity [62]. 

In studies conducted on zebrafish (*Danio rerio*), telomeres of gut and muscle cells were becoming shorter with age, which correlated with the formation of persistent DDR foci. Simultaneously with the growing number of short telomeres, fish began to develop age-associated conditions, such as gut inflammation and epithelium atrophy, which progressed with age, as well as myocyte degeneration (44% less muscle mass in 36-month-old in comparison to 3-month-old zebrafish) [63]. The relationship of telomere length in leukocytes and mortality was inversely proportional (during 16 years since beginning of the study). The study was conducted on a group of 1978 people aged 60 to 101 years. A correlation between telomere length and dementia was also demonstrated [60]. Some of the diseases that cause premature aging, i.e., Werner syndrome, are characterized by excessive shortening of telomere length [64]. Telomeropathies are a group of disorders resulting among many other symptoms in very short telomere length and disease anticipation. They are caused by a loss of function in genes required for telomere maintenance [65,66]. The most severe telomeropathies are dyskeratosis congenita, pulmonary fibrosis and aplastic anaemia [67]. It can result in premature ageing and high probability of cancer or leukaemia development (see [65,66,68,69] for review).

The amount of TAF foci increases with age. Comparing airway epithelial cells in 6,5-24-month-old mice revealed an increase in the number of cells containing TAF foci and increased the amount of TAF foci in cells. These cells exhibited high telomerase activity; hence, telomere attrition was not the cause of ageing in that instance. Symptoms of emphysema (a decreased number of pulmonary alveoli and their enlargement) can be observed quite often in ageing lungs. An inverse relationship between the amount of TAF foci and the number of alveoli was observed in ageing mice. It may suggest that telomere damage is involved in lung ageing [70]. 

Telomerase can influence survival rates and may be useful in the undoing of age-derived tissue degeneration. Mice with knock-out TERT showed signs of tissue atrophy, and their median survival decreased from 86.8 to 43.5 weeks. TERT reactivation resulted in the disappearance of a large amount of DDR foci, the regeneration of tissues with high a proliferative potential, and the restoration of brain mass from 77.3 ± 3.3% to 89.7 ± 4.0% [71]. 

Another factor that offers the ability to influence telomere length and, thereby, cell ageing is sirtuin 6 (SIRT6). Ge et al. [72] noticed a decrease in SIRT6 levels in oocytes of aged mice (42 to 45 months old). Sirtuin 6 expression was approximately 60% lower than that of oocytes in young mice. Two-cell embryos derived from oocytes with knocked-out SIRT6 had significantly shorter telomeres and their development was impaired. In contrast, SIRT6 overexpression caused telomere elongation. It is presumed that these disruptions are caused by an altered telomere chromatin structure—SIRT6 is responsible for the acetylation of histones H3K56 and H3K9 in mitotic cells [72]. Although studies on mice may be convenient and can provide insights into the process of ageing and telomere shortening, it must be noted that telomeres and telomere damage signalling are different in mouse and human cells, so conclusions from studies on mice cannot be automatically applied to humans [33].

### 3.2. Accumulation of Senescent Cells and Organismal Ageing

The accumulation of senescent cells in tissues can contribute to the process of organismal ageing [1]. Some components of SASP have the ability to make other cells senescent. This phenomenon is called the bystander effect. Young fibroblasts placed in close proximity to old fibroblasts had an increased number of DDR foci after two days of exposure. In the following ten days, the number of persistent DDR foci in young fibroblasts was comparable to that of senescent fibroblasts [73].

Tissues of ageing organisms are characterised by a larger number of senescent cells than tissues of young organisms [1,43]. The skin of ageing baboons had elevated levels of HIRA in over 70% of fibroblasts (which is typical of senescent cells). Young baboons had elevated HIRA levels in less than 20% of cells. These changes were not detected in myocytes. This shows that the number of senescent cells also depends on the type of tissue [1].

A portion of SASP proteins secreted by senescent cells is proinflammatory proteins. They can be one of the factors causing chronic inflammation present in aged organisms [74]. Studies on healthy Caucasians and African-Americans demonstrated an increase in systemic inflammation with age, regardless of the ethnic group [75]. 

Chronic inflammation takes place when there is a prolonged increased concentration of proinflammatory factors in the bloodstream. It can lead to tissue fibrosis or necrosis and promote several age-related diseases, e.g., neurodegenerative diseases (Alzheimer’s, Parkinson’s), cardiovascular diseases (atherosclerosis, cardiomyopathy), metabolic disorders (type II diabetes, sleep apnoea, fatty liver disease), musculoskeletal disorders (osteoporosis, osteoarthritis, sarcopenia) and cancer [76].

In studies on centenarians and semi-supercentenarians, inflammation was the second most important factor (after age) that determined survivability, as well as physical and psychological prowess [77]. 

**Figure 2:**
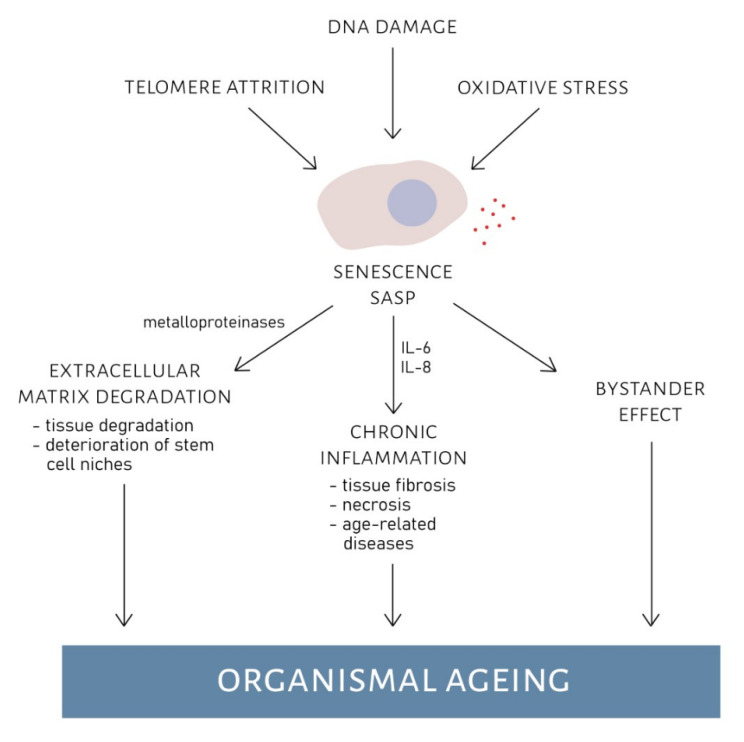
Effects of senescence-associated secretory phenotype (SASP) on organismal ageing. Telomere attrition, DNA damage or oxidative stress can cause the cell to enter senescence. Senescent cells can develop SASP. Substances that are excreted during SASP can have detrimental effect on extracellular structures and neighbouring cells. Metalloproteinases cause extracellular matrix degradation leading to deterioration of tissues and stem cell niches. Inflammation factors (e.g., IL-6, IL-8) might cause chronic inflammation, that can lead to tissue fibrosis, necrosis and age-related diseases. Finally, SASP has a bystander effect on neighbouring cells, causing them to enter senescence. All these effects propagate organismal ageing.

Xu et al. [78] managed to decrease inflammation in mice adipose tissue by JAK inhibition, which led to SASP suppression. Pleiotropic JAK/STAT pathway (Janus kinase/signal transducers and activators of transcription) is responsible for transmitting signals to various cytokines and growth factors. Its activation stimulates cell proliferation, differentiation, migration, and apoptosis [79,80,81]. From 15 to 50% of adipose tissue cells are preadipocytes (precursors of adipocytes). An investigation into the quantity of senescent preadipocytes in young and old subjects revealed their accumulation during ageing. SASP factors secreted by those senescent preadipocytes caused adipose tissue inflammation [78]. Potential effects of SASP on organismal ageing are shown in Figure 2.

P16^INK4A^ protein can push cells into senescence by blocking RB activation. Expression of p16^INK4A^ is increased during ageing; hence, it is thought that it could be used as a biomarker of biological ageing [82]. However, Hall et al. [83] suggests that some of the cells accumulated in tissues that were thus far considered to be senescent (on account of p16^INK4A^ and β-galactosidase presence) are actually macrophages which express p16^INK4A^ and β-gal in pH 6. The majority of adipose tissue cells of aged mice marked previously as senescent by means of β-gal detection turned out to be macrophages instead. This leads to a conclusion that not only senescent cells but also macrophages are involved in the propagation of inflammation and ageing [83]. Metalloproteinases secreted by senescent cells cause extracellular matrix degradation. This can lead to the deterioration of stem cell niches. Matrix degradation can also bring about the loss of skin and lung elasticity [43].

Clearing senescent cells from tissues can have therapeutic effects on ageing organisms. In a study on mice, senescent cells were pushed into apoptosis by FOXO4 inhibition. FOXO4 is a transcription factor that suppresses the initiation of apoptosis in senescent cells. Targeting senescent cells of aged mice for apoptosis resulted in improved fur condition, increased physical activity and improved kidney function (indicated by a lower concentration of urea and creatinine in plasma). IL-6 expression in kidneys was reduced and lamin B1 levels increased [84].

### 3.3. Changes in Chromatin Structure and Organismal Ageing

Epigenetic modifications can influence the length of life and the ageing process [85,86].

Retaining adequate amounts of heterochromatin in the nucleus is vital for cellular functioning. Heterochromatin is necessary for genome stability. It participates in maintaining the structure of the nucleus. Reducing the amount of heterochromatin by halving the expression of HP1 (heterochromatin protein 1 which is crucial for keeping chromatin condensed) shortened the lifespan of *Drosophila melanogaster* considerably. Mildly increasing HP1 expression amplified median survival rates by 23%; however, a significantly higher expression led to developmental anomalies and death. A gradual heterochromatin loss occurs naturally during the process of ageing. Erythrocytes of aged drosophila had much lower levels of heterochromatin than erythrocytes of young fruit flies. This is also the case for people afflicted with various kinds of premature ageing disorders [87].

DNA methylation is a postreplicative modification of DNA which plays an important role in regulation of gene expression during normal developmental stages of organisms, as well as in ageing [88]. The most often methylated nitrogen base in DNA is cytosine [89,90]. Clusters of these nucleotides called CpG islands are abundant in the promoter and regulatory regions of genes. Methylation of the CpG islands in the promoter region leads to gene silencing [91]. In humans, only 20–30% of promoter regions are not methylated, which is related to transcriptionally active genes [92]. DNA methylation levels change with increasing age [93,94]. Comparing CD4^+^ T lymphocytes of new-borns and centenarians revealed methylation changes in promoters (~10%), exons (~10%), intrones (~45%), and intergenic regions (~35%). Although we observe a global decrease in DNA methylation in the ageing process, this does not apply to all genes. In some of them, we observe the opposite process [86]. In addition, the methylation level of both individual genes and the general level is tissue and individually specific [95,96,97]. The progress in the study of the dependence of the methylation degree of specific loci in the genome on the chronological and biological variation allows for the assumption that in the future it will be possible to develop models predicting the life expectancy and biological age of a human based on the methylation degree of specific loci in selected tissues [98,99,100,101]. It can be achieved by creating methylation profiles of several selected loci. Analysis of methylation of these markers in samples collected from people of various ages can become a basis for constructing age-predictive models [99]. The creation of these models can be realized by using statistical and machine-learning algorithms [98,100]. 

Ageing is accompanied by global histone loss. This is thought to be caused by their deposition, which is regulated by ASF1 protein, as well as CAF1 and HIRA. ASF1 expression is diminished during ageing. The quantity of different histone variants is also subject to change (Table 1) [86].

The effects of changes in histone methylation that were passed on to the next generations can become apparent for the first time even several generations after initial modification. In studies conducted on *Caenorhabditis elegans*, the deletion of gene encoding SPR-5 (histone H3K4me2 demethylase) increased the lifespan of modified *C. elegans* specimens but only beginning from the seventh or eighth generation. The effect was maintained up to the twentieth generation [85].

Sirtuin 6 (SIRT6) is a chromatin regulator responsible for the deacetylation of H3K9ac, H3K56ac and H3K18ac histones (among others). Owing to this, SIRT6 can impact the level of chromatin condensation and transcription suppression. It participates in the repression of genes that encode chromatin regulators (e.g., NF-κB and HIF-1) that are suspected of contributing to tumour development and ageing. Sirtuin 6 alters chromatin structure in response to DNA damage and facilitates its repair. Furthermore, it assists telomeres in retaining their shape. Decreasing SIRT6 levels in mice caused lifespan reduction, as well as led to phenotypic changes associated with ageing and neoplastic transformation [102].

**Figure 3:**
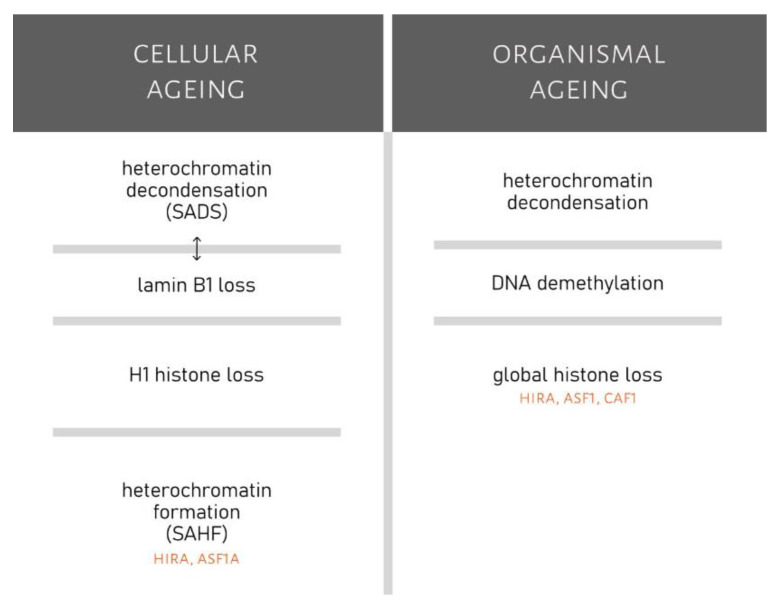
Comparison of chromatin changes during cellular and organismal ageing. Changes in chromatin of senescent cells include chromatin decondensation (SADS), lamin B1 loss (which could be connected to SADS), loss of histone H1 and formation of heterochromatin that is propagated by HIRA (histone repressor A) and ASF1a (antisilencing function protein 1a). Chromatin changes linked to organismal ageing are heterochromatin decondensation, DNA demethylation and global histone loss (which might be propagated by HIRA, ASF1 (anti-silencing function protein 1) and CAF1 (chromatin assembly factor 1)).

Summarized changes in chromatin that occur during cellular and organismal ageing are displayed in Figure 3.

## 4. Conclusions

1. Telomeres became shorter with age, which correlated with the formation of persistent DDR foci.
2. Impossible to repair persistent DDR foci can cause the cell to go into a state of senescence.
3. Telomere associated persistent DDR foci are one of the causes of ageing of differentiated or quiescent cells.
4. Shortening of telomeres to a critical length under 6000–8000 base pairs results in disconnection of the telomere protective shelterin and formation of multiple non-repairable double-stranded breaks leading to senescence.
5. Shortening of telomeres can be inverted by activating TERT and SIRT6 genes.
6. Secretion of proteins by senescent cells can affect neighbouring and distant cells of the body by stimulating or inhibiting proliferation.
7. Senescence-associated heterochromatin foci alter the transcriptome of the cell.
8. The accumulation of senescent cells in tissues can contribute to the process of organismal ageing.
9. Clearing senescent cells from tissues can have therapeutic effects on ageing organisms.
10. The global demethylation of DNA is a hallmark of ageing.
11. Methylation profiling is a good way to accurately estimate the biological age in humans.

## Figures and Tables

**Table 1:** Changing amounts of histone variants with age [86]. Arrow pointing up—increased amount of histone; arrow pointing down—decreased amount of histone.

Histone Variant	Yeast	Mouse	Rat	Human
H3K9me				↓
H3K9me3		↓		↑
H3K9ac		↓	↑	↓
H3K56ac	↓			↓
H3.1	↑		↑	↑
H3.3			↓	↓
H2A.1	↑		↑	↑
H2A.2			↓	↓
γ-H2AX		↓		↓

## Data Availability

Data sharing not applicable.

## Abbreviations

γ-H2AX	histone H2AX phosphorylated on Ser139
53BP1	p53-binding protein 1
alt-NHEJ	alternative non-homologous end-joining
ASF1	anti-silencing function protein 1; histone chaperone ASF1
ASF1a	antisilencing function protein 1a; histone chaperone ASF1
ATM	ataxia telangiectasia mutated protein; serine-protein kinase ATM
ATR	ATM-and RAD-3-related kinase
BJ	human fibroblasts cell line
CAF1	chromatin assembly factor 1
CCL	chemokine (c-c motiff) ligand
Chk1	serine/threonine-protein kinase 1; checkpoint kinase 1
Chk2	serine/threonine-protein kinase 2; checkpoint kinase 2
c-NHEJ	canonical non-homologous end-joining
DDR	DNA damage response
DSB	DNA double-strand breaks
E2F	group of genes that encodes a family of transcription factors
FOXO4	forkhead box protein O4
GRO	growth-related oncogene
H2A.1	histone H2A.1
H2A.2	histone H2A.2
H2AX	variant of H3.1 histone
H3.3	histone H3.3
H3K4me2	histone H3 methylated on lysine 4
H3K56ac	histone H3 acetylated on lysine 56
H3K9ac	histone H3 acetylated on lysine 9
H3K9me	histone H3 methylated on lysine 9
H3K9me3	histone H3 triple methylated on lysine 9
HGF	hepatocyte growth factor
HIF-1	hypoxia inducible factor 1
HIRA	histone repressor A
HP1	heterochromatin protein 1
HR	homologous recombination
IGFBP	insulin-like growth factor binding protein
IL-6	interleukin 6
IL-8	interleukin 8
IMR90	Institute for Medical Research-90 cell line; human fetal lung fibroblast cell line
Ki-67	proliferation marker protein Ki-67
LMNB1	gene encoding lamin B1
MDA231	breast cancer cell line
MDC1	mediator of DNA damage checkpoint protein 1
MRC-5	fetal lung cell line
MRE11	double-strand break repair protein MRE11
MRN	MRE11/RAD50/NBS1 complex
NAD+	nicotinamide adenine dinucleotide
NFBD1	nuclear factor with BRCT domains protein 1
NF-κB	nuclear factor kappa B
NHEJ	non-homogenous end joining
p51	p51 protein
p53	p53 protein
PARP1	poly (ADP-ribose) polymerase
POT1	protection of telomeres 1
RAD17	cell cycle checkpoint protein RAD17
RAD50	RAD50 double strand break repair protein
Rap1	repressor-activator protein 1
Rb	retinoblastoma
ROS	reactive oxygen species
RPE	retinal pigment epithelium
SADS	senescence-associated distension of satelites
SAHF	senescence-associated heterochromatin foci
SASP	senescence-associated secretory phenotype
SIRT1	sirtuin 1
SIRT6	sirtuin 6
SPR-5	probable lysine-specific histone demethylase 5
TAF	telomere associated foci
TERC	telomerase RNA component
TERT	telomerase reverse transcriptase
TIN1	tunicamycin induced protein 1
TPP1	tripeptidyl peptidase 1
TRF2	telomere repeat binding factor 2
WI-38	human diploid lung fibroblasts cell line
